# Proton Pump Inhibitors and Bone Health: An Update Narrative Review

**DOI:** 10.3390/ijms231810733

**Published:** 2022-09-14

**Authors:** Eric Lespessailles, Hechmi Toumi

**Affiliations:** Department of Rheumatology, Translational Medicine Research Platform, PRIMMO, Regional Hospital of Orleans, 45067 Orleans, France; hechmi.toumi@univ-orleans.fr

**Keywords:** bisphosphonates, bone mineral mensity, fall, fracture risk, osteoporosis, proton pump inhibitor

## Abstract

Proton pump inhibitors (PPIs) are an antacid drug often used in acid-related disorders. They decrease acid secretion in the stomach by blocking an enzyme called H+/K+ ATPase which controls acid production. Introduced to the market in 1989, their use has increased rapidly worldwide and they are now among the top 10 most prescribed drugs in the United States. As of 2015, the FDA has already approved six drugs of this class (omeprazole, esomeprazole, lansoprazole, dexlansoprazole, pantoprazole and rabeprazole). Recently, the risks and benefits of long-term PPI use were questioned and many studies indicated that their use should be carefully considered, especially in young patients, whose treatment with these drugs could last many years. Even greater concerns have been raised about a potential positive association between PPIs and osteoporotic fracture risk including the hip, spine and wrist. Although based on observational studies, there is substantial evidence associating the long-term use of PPIs and fracture. This relationship is only partially admitted due to the lack of consistent effects of PPIs on bone mineral density loss. Therefore, this narrative review aimed to discuss the recent findings pertaining to the risk of osteoporotic fracture associated with PPIs, in particular prolonged use, and to call for further research to elucidate the mechanisms associated with this bone fragility.

## 1. Impact Statement

Proton pump inhibitors (PPIs) are among the most widely prescribed drugs. However, their safety with respect to bone health is debated. Their use has risen recently, particularly in the older population which is also affected by bone fragility. This narrative review explores different facets aspects of the biological plausibility of this concern. The review underlines the paucity of relevant studies explaining the bone fragility associated with PPI use, as bone mineral density (BMD) loss does not seem to be a primary contributor. This study raises concerns regarding the risk of osteoporotic fracture associated with prolonged PPI use and calls for further research to elucidate the mechanisms associated with this bone fragility.

## 2. Introduction

Proton pump inhibitor (PPI) therapy has profoundly changed the treatment of acid-related gastrointestinal diseases and their related consequences such as gastroesophageal reflux disease, peptic ulcer disease and dyspepsia but also the prevention of gastric ulcers and bleeding or the use of prophylactic drugs among users of non-steroidal anti-inflammatory drugs [1].

Thus, PPIs are widely prescribed worldwide [2,3]; a nation-wide observational study has found as many as 16 million prescriptions in 2015 in France [4]. A study using data of one German statutory health insurance company from 2005 to 2013 found that the PPI prescribing prevalence increased from 8.2 to 16.2%. [5]. However, besides their appropriate use in gastrointestinal conditions, PPIs are among the most frequently sold over-the-counter agents [6,7,8].

Although PPIs are considered to be a safe medication for relevant recommended therapeutic use, a number of undesirable related effects have been described including clostridium difficile-associated diarrhea [9], community-acquired pneumonia [10], but also possibly some cancers induced by intestinal dysbiosis [11]. Recently, using an electronic medical record database from general practitioners in the United Kingdom, it was concluded by the authors that initiation of pantoprazole or omeprazole use was associated with a higher risk of knee replacement than initiation of histamin-2 receptor antagonist [12]. Additionally, increasing concerns have been raised about the potential adverse effect of PPIs on bone health [13,14,15].

The aim of this narrative review was to summarize and update current knowledge on the effects of PPI use on bone health. We also explore the plausible biological mechanisms for the association between PPIs and bone metabolism. Table 1 provides an overall summary of the potential effects of PPI on bone status.

## 3. Overall Biological Plausibility for the Association between Fragility Fracture and PPI Use

As randomized controlled trials designed to specifically identify the risk of low energy fracture in populations when treated with long-term PPIs are lacking, the likelihood of causality is based on observational studies. One of the plausible biological mechanisms proposed is a decreased intestinal absorption of calcium which in turn leads to a negative calcium balance resulting in secondary hyperparathyroidism with an increase in bone loss and fractures [16,17]. However, the results of the effects of PPIs on calcium absorption are inconsistent [17,18,19]. Besides their effects on calcium, IPP might also affect phosphorus as a rat study indicated that PPI administration influenced phosphorus metabolism via a higher phosphorus absorption [20].

Other interaction between the effects of PPI and diet have been evidenced in rats [20,21]. In vitro studies have shown that PPIs, namely omeprazole, inhibited bone resorption [22,23] via selective inhibition of osteoclasts vacuolar H^+^-ATPase [24]. Omeprazole, an inhibitor of H^+^, K^+^-ATPase was used to assess the possibility of the suppression of bone resorption by PPIs in humans [25]. These findings echoed with another experimental study in ovariectomized rats in whom the administration of a selective inhibitor of the osteoclastic vacuolar-type H+-ATPases suggested that PPIs might prevent osteoporosis [26]. Interfering with the function of osteoblast cell activity, it was further demonstrated that PPIs had a concomitant and dose-dependent inhibitory effect on human osteoclast [27].

Other possible explanations for an effect of PPIs on bone fracture and bone metabolism include PPI-induced hypergastrinemia resulting in parathyroid hypertrophy or hyperplasia [28]; impaired absorption of vitamin B12 [29,30,31] might also interfere with homocysteine level which in turn may contribute to the susceptibility of fracture in PPI users through alteration in the quality of collagen. [32,33].

Lastly, an alternative explanation is the role of long-term PPI use on the gut microbiome through several alterations including hypomagnesemia [34,35,36,37] and hypocalcemia [38]. PPI use is associated with low magnesium status [39] and a recent comprehensive review has reported that low magnesium levels induced by PPI were related to decreased muscle function [40]. Consequently, hypomagnesemia may affect bone health through its role on both relevant actors of the bone metabolism, i.e., calcium and vitamin D as magnesium play a role as a natural calcium antagonist and plays a key role in vitamin D metabolism as 25-hydroxylase and 1-α hydroxylase are both magnesium-dependant [41].

## 4. PPI Use and Bone Mineral Density

Among the mechanisms that could lead to an increased risk of fracture associated with long-term PPI use, it has been hypothesized that PPIs could alter intestinal calcium absorption, thus resulting in increased rates of bone loss. Consequently, investigating potential BMD induced changes associated with PPI use is a relevant task.

### 4.1. Animal Studies

The first animal study investigating the potential causal effect of the long-term effects of PPIs on BMD identified a decrease in BMD among young male rats treated for 77 days at a dose of 400 µmole/kg of omeprazole [42].

In another experiment, during 90 consecutive days, 50 Wistar rats received either different doses of omeprazole (300 µmole/kg) or the dilution vehicle in order to assess femoral BMD by Dual Energy X-ray Analysis (DXA) and bone biomechanical properties by a three-point mechanical flexion test. Mean femoral BMD was significantly lower in the group with the highest dosage of omeprazole as compared to the control group. However, mechanical properties were not altered whatever the dose used in the study. These findings are echoed in another experiment which was carried out on young male Wistar rats receiving either pantoprazole at a dose of 3 mg/kg or vehicle for 12 weeks [43]. A significant decrease in both femoral BMD and in growth plate thickness was detected in pantoprazole-treated animals [43].

### 4.2. Human Studies

BMD reduction associated with PPI use was investigated in well-known cohort studies such as the study of osteoporotic fractures (SOF), the osteoporotic fractures in men study (MrOs) and the women’s health initiative (WHI) [44,45,46] studies. In none of these studies were longitudinal BMD changes affected by PPI exposure. Using the Manitoba BMD database the authors concluded that PPI use did not appear to be associated with osteoporosis or with accelerated BMD loss [47]. These data are consistent with the findings from the SWAN study showing no association between PPI use and BMD loss [48].

Several recently published systematic reviews have addressed the question of the potential BMD loss associated with the prolonged use of PPIs [15,49,50]. Although based on a limited number (6) of prospective or retrospective cohorts, the conclusion was always the same: there were no significant changes in BMD between PPI users and non-users [15,49,50]. In the meta-analysis by Aleraij et al., there was however a statistically significant reduction in the mean BMD difference among PPI users (−0.03; 95% CI −0.04, −0.01).

These contradictory results do not permit any relevant conclusions. It should be borne in mind that even the best meta-analyses conducted with a rigorous methodology have inherent limitations based on the limited number of studies included the lack of randomized control trials, the age variations in the populations studied (i.e., mean age range from 37.7 to 79.3 in [50]) or other potential confounding factors not evaluated in the study such as types of PPI use, study design, sample size and exposure duration that could lead to significant heterogeneity in the outcomes.

## 5. PPI Use and Bone Quality

A number of studies listed below (Section 6) have shown a significant association between PPI use and bone fractures, particularly vertebral fracture (VF). Intriguingly, the mechanisms underlying this relationship are not elucidated.

### 5.1. Animal Studies

The effect of pantoprazole on bone healing was assessed in aged mice using a rodent fracture model [51]. A daily intraperitoneal injection of 100 mg/kg body weight pantoprazole or vehicle was administered to aged mice. It was concluded that pantoprazole impaired fracture repair; µCT analyses showed that trabecular thickness was significantly reduced in the treated animals compared to controls. However, in this fracture healing study, pantoprazole therapy did not induce a significant difference in the bending stiffness of the non-fractured contralateral femoral when compared to controls [51].

Hypothesizing that the bone effects of PPIs might be related to the inhibition of the bone-specific phosphatase PHOSPHO1 and matrix mineralization, an in vitro experiment was investigated [52]. Contrasting with the lack of inhibitory effects of the histamine-2 receptor antagonists on PHOSPHO1 activity, all PPIs tested inhibited the activity of PHOSPHO1 [52]. Additionally, in a concentration dependent manner, the mineralization of bone matrix in primary osteoblast culture was inhibited by several PPIs [52].

Another animal study conducted in three-month-old Sprague Dawley male rats found a significant deterioration of trabecular bone microstructure associated with the use of pantoprazole 3 mg/kg per os during 60 days but neither bone cellular histomorphometry nor bone biomechanical properties were affected [53].

### 5.2. Human Studies

Most of the studies assessing bone loss associated with PPI use in humans measured this potential loss by DXA Technology [54,55] and failed to demonstrate a clear association. However, DXA has limitations in its ability to separate the trabecular and cortical bone compartments and evaluate only areal BMD but not volumetric BMD even though such limitations can be ruled out when using High-Resolution peripheral Quantitative Computed Tomography (QCT) or peripheral (pQCT) devices [56,57,58]. To the best of our knowledge, no studies have yet investigated the effects of PPI use on bone microarchitecture. However, in a cross-sectional study, a small sample of elderly patients was investigated through the use of pQCT permitting differential assessment of cortical and trabecular volumetric density. After adjustment for sex and age, it was found that trabecular bone density was lower in PPI users than in non-users. Interestingly, bone geometry and cortical bone density parameters were not affected by PPI therapy [28].

Trabecular bone score (TBS), which is a validated texture parameter indirectly related to trabecular microarchitecture [59], was used to investigate the influence of PPIs on trabecular bone microarchitecture [60]. The medical records of 1505 women who had a DXA scan were reviewed and analyzed [60]. Findings showed a significantly lower TBS and BMD in the PPI exposure group as compared to the control group. Lower TBS was associated with current PPI use but not with past use, suggesting a reversible effect of PPIs on trabecular bone changes [60].

## 6. PPIs and Fragility Fractures

The seminal paper providing evidence that PPIs may increase the risk of VF reported a Danish case–control study showing that recent (use within the last year) PPI therapy was associated with an increased risk of VF in PPI users as compared to non-users (odds ratio: 1.60; 95% CI 1.25–2.04) [61]. Further evidence that PPI use could be associated with an increased risk of fragility fracture was reported in later prospective studies [62,63]. In the OsteoPorosis and Ultrasound Study, 1211 post-menopausal women were enrolled and assessed by X-rays at baseline and at the 6-year follow up to evaluate VF [62]. Five percent of the population was using omeprazole and a multivariate analysis showed that omeprazole use was a significant and independent predictor of VF (RR = 3.5; 95 CI 1.14–8.44) [62].

In a prospective study which included 161,806 postmenopausal women, results from the Women’s Health Initiative further confirmed that the use of PPIs was associated with clinical VF with a Hazard Ratio for current PPI use of 1.47 (95% CI, 1.18–1.82) [63]. Based on the Nurses’ Health Study, the association between long duration (>12 years) of PPI use and VF risk was investigated [14]. In this prospective study 55,545 women were assessed for VF that was confirmed by medical record review. Based on the data collected during 606,848 person years of follow up over a 12-year period, the multivariate adjusted relative-risk obtained showed that longer duration of PPI use was associated with higher VF risk. Additionally, after adjusting for age and history of osteoporosis, PPI use was still associated with an increased risk of VF (RR 1.44; 95% CI 1.17–1.77 and RR 1.29; 95% CI 1.04–1.59) [14].

Thus, based on these last two prospective studies, we can conclude that there is a consistent body of evidence (although based on observational studies) suggesting that PPI use could be associated with an increased risk of VF.

Furthermore, the increased risk of fragility fracture does not seem to be limited to VF.

An increased risk of hip fracture in users of PPI therapy was also evidenced using the General Practice Research Database (GPRD) [64]. In this nested case–control study the crude incidence rate of hip fracture was estimated to be 4.0/1000 person years among patients with PPI therapy and 1.8/1000 person years among acid suppression non-users [64]. In the Manitoba study [65], using an extensively validated population for assessing the prevalence of osteoporosis related fractures, it was found that there was a significant association between ≥7 years of continuous PPIs and vertebral, wrist and hip fractures. An association between hip fractures and 5 or more years’ exposure to PPIs was demonstrated (adjusted OR 1.62, 95% CI 1.02–2.58, *p* = 0.04). The longer the exposure, the higher the risk, with even higher risk (adjusted OR 4.55, 95% CI 1.68–12.29, *p* = 0.002) in patients with ≥7 or more years of PPI exposure.

However, it should be noted that using the United Kingdom General Practice Research Database, a nested case–control study, the relative risk for hip fracture was 0.9 (95% CI 0.7–1.1) among patients who received any PPIs versus those with non PPI prescriptions [66].

This lack of association was further confirmed in another older adult cohort where the data suggested that no significant association could be detected between PPI therapy and fracture risk [67].

Conversely, in the pooled analysis of the well phenotyped (MrOS) and SOF studies, an increased risk of non-spine fracture was found in women using PPIs (RH = 1.34, 95% CI 1.10–1.64) [68]. In MrOS, an increased risk of non-spine fracture in men was also found but only among those who did not take calcium supplements (RH = 1.49, 95% CI 1.04–2.14) [68]. In the Nurses’ Health Study the authors examined the association between long-term PPI use and risk of hip fracture [69]. Findings demonstrated that PPI use was associated with a 50% increase in fracture risk in current and former smokers although no association was found in non-smokers [69].

Taking the aforementioned studies and others into account, the US Food and Drug administration modified the labeling for PPIs in 2010 to include information about the potential risk of vertebral, hip and non-hip, non-vertebral fractures [70].

Based on a data mining set from the Food and Drug Administration Adverse Event Reporting System, results confirmed that PPI use appeared to be associated with an increased risk for fractures at multiple sites [71].

Several meta-analyses and systematic reviews have been published focusing on the risk of fractures associated with the use of PPIs [15,49,72,73,74].

## 7. Effect of Bisphosphonates on PPI-Induced Fracture Risk

PPIs are commonly prescribed to patients treated with bisphosphonates for osteoporosis management due to upper gastrointestinal adverse effects [75].

Many studies have identified that patients on bisphosphonates who are on PPIs have a further increased risk of fracture as compared to patients receiving bisphosphonates alone [76,77,78]. In a post hoc analysis of a subset of patients stemming from three randomized clinical trials which evaluated the anti-fracture efficacy of risedronate, the authors explored the relationship between concomitant PPI use and incident fractures [79]. Findings showed that risedronate reduced the risk of new vertebral fractures regardless of concomitant therapy with PPIs [79]. Conversely, in both a Korean population-based study and a Spanish cohort, it was found that PPI use was associated with fracture in bisphosphonate users [77,80]. Hence, after stratification according to bisphosphonate use, OR for hip fracture was increased to 1.71 (95% CI 1.31–2.23) in patients taking bisphosphonates and PPIs while it was 1.30 (95% CI 1.19–1.42) in bisphosphonate non-users. Using data from a Spanish public health-care system representative of the population of Catalonia, the authors identified predictors of “fracture while on treatment” in people starting bisphosphonate therapy. In this cohort, with accurate data on both persistence and compliance, 60% of the patients taking bisphosphonates also used PPIs. It was demonstrated that use of PPIs constituted one of the key factors associated with increased risk of fractures even when adherence and persistence were respected [80]. Taken together these data suggest a potential interaction between bisphosphonates and PPIs that may explain the mechanism for increased fracture risk [77].

In the vertebral fracture treatment comparisons in osteoporotic women (VERO) study, a head-to-head trial comparing risedronate (an anti-resorptive therapy) to teriparatide (a bone-forming drug) in the prevention of fractures, it was concluded by the authors that in the whole population, patients receiving PPIs had a higher risk for fractures compared to those not on PPIs [81]. However, the fracture efficacy results of teriparatide versus risedronate did not significantly differ within the categories of PPIs [81]. Finally, using a Danish national register-based cohort study the authors demonstrated that concurrent PPI use was associated with a dose-dependent loss of protection against hip fracture with alendronate in elderly patients [82] suggesting a blunting of the anti-fracture efficacy of alendronate (half of the effect was lost).

## 8. PPI Use and Falls

The primary risk factor for non-vertebral fracture, and hip fracture particularly, is a fall; as many as 90% of all fractures occur after a fall [83]. Consequently, another explanation for the increase in fracture risk with prolonged PPI use could be an increase in the rate of falls. A substantial proportion of fractures occur in subjects with T-scores which do not reach the osteoporotic threshold [84,85,86,87] and an increased rate of fall is often considered as the main cause of the fracture in these patients. Furthermore, there are several leads to associate long-term use of PPIs and risk of falls. Among the factors which were reported in patients on long-term PPI therapy appear visual impairment, vitamin B12 deficiency or their neurologic associated consequences such as numbness of the feet [88,89,90,91].

In a post hoc analysis, but with replication of the findings in a separate study in which falls were the primary outcome, it was identified that elderly postmenopausal women had an increased risk of falls and fracture-related hospitalizations with long-term PPI use [89]. Other authors identified an increased risk of falls associated with PPI use in older women [92]. Findings from the VERO trial also supported this hypothesis, as patients taking PPIs reported a higher frequency of falls as compared to non-users, although the difference did not reach the level of statistical significance [81].

## 9. Conclusions

Given the widespread use of PPIs, reinforced by frequent inappropriate indications, physicians should be aware that long-term PPI use is associated with a modest but rather consistent increase in the risk of fragility fracture. Consequently, long term PPI use should be prescribed only in cases of strong validated and recognized indications.

Their responsibility in the increase of fracture risk, even though considered as modest, should be taken into consideration in the management of elderly patients at risk of fragility fracture.

The effects of PPIs on bone homeostasis and the mechanisms explaining their effect on fracture risk are far from being elucidated. Recent emergent potential causes such as trabecular bone alterations, disturbances in matrix mineralization and effects on bone-specific phosphatase need further well-conducted trials to confirm their role in PPI-related bone damage.

## Figures and Tables

**Table 1:** Overview of the different effect of proton pump inhibitors on bone health. BMD: bone mineral density, DXA: dual energy X-ray analysis, Micro CT: micro computed tomography, pQCT: high-resolution peripheral quantitative peripheral.

PPI	Animal Studies	Human Studies
**Biological plausibility**	Increase of bone resorption Decrease of osteoclast vacuolar V^+^-ATPase	Decrease of osteoclast vacuolar H^+^-ATPase Hypergastrinemia Decrease of quality of collagen Increase of bone resorption
**Bone Mineral Density (DXA)**	Decrease of femoral BMD	Inconsistent data
**Bone quality**	Decrease of trabecular thickness (micro CT)	Decrease of trabecular bone density (pQCT) Decrease of Trabecular Bone Score (DXA)
**Frequency of falls**	-	Increased risk of fall
**Epidemiological data on fragility fracture**	-	Increased risk of fragility fracture

## Data Availability

Not applicable.
